# COVID-19 Vaccine: Predicting Vaccine Types and Assessing Mortality Risk Through Ensemble Learning Agorithms

**DOI:** 10.12688/f1000research.140395.1

**Published:** 2023-09-25

**Authors:** Hind Monadhel, Ayad R. Abbas, Athraa Jasim Mohammed

**Affiliations:** 1 Computer Science, University of Technology - Iraq, Baghdad, Iraq; 2 Computer Science, University of Technology- Iraq, Baghdad, Iraq; 3 Computer Science, University of Technology- Iraq, Baghdad, Iraq

**Keywords:** Classification algorithm, COVID-19 Vaccine, ensemble learning, machine learning, Sampling methods, Side effects.

## Abstract

**Background:** There is no doubt that vaccination is crucial for preventing the spread of diseases; however, not every vaccine is perfect or will work for everyone. The main objective of this work is to predict which vaccine will be most effective for a candidate without causing severe adverse reactions and to categorize a patient as potentially at high risk of death from the COVID-19 vaccine.

**Methods:** A comprehensive analysis was conducted using a dataset on COVID-19 vaccine adverse reactions, exploring binary and multiclass classification scenarios. Ensemble models, including Random Forest, Decision Tree, Light Gradient Boosting, and extreme gradient boosting algorithm, were utilized to achieve accurate predictions. Class balancing techniques like SMOTE, TOMEK_LINK, and SMOTETOMEK were incorporated to enhance model performance.

**Results:** The study revealed that pre-existing conditions such as diabetes, hypertension, heart disease, history of allergies, prior vaccinations, other medications, age, and gender were crucial factors associated with poor outcomes. Moreover, using medical history, the ensemble learning classifiers achieved accuracy scores ranging from 75% to 87% in predicting the vaccine type and mortality possibility. The Random Forest model emerged as the best prediction model, while the implementation of the SMOTE and SMOTETOMEK methods generally improved model performance.

**Conclusion:** The random forest model emerges as the top recommendation for machine learning tasks that require high accuracy and resilience. Moreover, the findings highlight the critical role of medical history in optimizing vaccine outcomes and minimizing adverse reactions.

**Abbreviations**
COVID-19: Coronavirus Disease 2019
DT: Decision Trees
LGBM: Light Gradient Boosting Machine
ML: Machine Learning
mRNA: messenger ribonucleic acid
RF: Random Forests
SARS-CoV: Severe Acute Respiratory Syndrome- associated coronavirus
SMOTE: Synthetic Minority Oversampling Technique
VAERS: Vaccine Adverse Event Reporting System
XGB: extreme Gradient Boosting Machine

## Introduction

From seven to 13 years of research and development (R&D) and 1.8 million clinical trials to develop a vaccine in the past, we have transitioned to 10 to 18 months of R&D and tens of thousands of clinical trials to start vaccinating against COVID-19 in 2021. ^1^

Vaccines are biologics that provide active adaptive immunity against particular diseases. The vaccine usually contains drugs similar to the microorganisms that cause the disease. It is generally made from one of the killed or attenuated micro-organisms, its toxins, or its surface proteins. Giving us an injection, nasal spray, or oral vaccine stimulates our immune system to recognize and destroy foreign bodies. ^2^

As a result of the novel coronavirus's rapid dissemination and disease burden, pharmaceutical companies and researchers were forced to create vaccinations quickly using either novel or preexisting technologies. ^3^ There are several different types of vaccines, and the purpose of each type is to boost your immune system and prevent serious, life-threatening diseases from occurring. ^4^ The COVID-19 vaccines that have been approved employ a variety of mechanisms of action, including mRNA, DNA vaccines, viral vectors, protein subunits, and virus-inactivated vaccination techniques. ^5^ Three vaccines have been widely administered: Pfizer and Moderna (mRNA) vaccinations targeting the SARS-CoV-2 surface protein, and the Janssen (viral vector) vaccine, which employed pre-existing technology with an adenovirus vector to trigger an immune response and provide protection against further infection. As these vaccines were developed using various approaches, they differ in efficacy and storage conditions. ^6^

However, no vaccine is entirely free from complications or adverse reactions. Any vaccination can have early adverse reactions, including local ones like pain, swelling, and redness, as well as systemic ones like headache, chills, nausea, fatigue, myalgia, and fever. ^7^ Also, several existing health conditions or symptoms the candidate already has can lead to severe adverse reactions after taking the COVID-19 vaccine. The candidate's death could be the worst-case scenario. As a result, it's critical to know about the candidate's previous medical history. ^8^

This work's main contribution can be summarized as follows:

1. Identify the most important features of an individual's medical history that could contribute to adverse reactions to vaccination.
2. Identify the most important features that contributed to the death of the candidate based on his or her medical history.
3. Address the challenge of the imbalanced dataset by employing sampling methods to effectively handle the imbalance and improve the reliability of the analysis.
4. Develop a machine learning (ML) model capable of predicting and classifying the most suitable vaccine types for each candidate, thus helping to prevent severe consequences and ensure optimal vaccination outcomes.

The rest of this paper is organized as follows: In the next section, we discuss a brief review of the literature on various related works. Section 3, provides a detailed explanation of our methodology and dataset. In Section 4, we discuss the study findings. while Section 5 discusses conclusions and potential future research.

## Literature review

Due to the rapid advancement of technology, there are numerous opportunities and possibilities for ML in healthcare. ^9^ Classification is the most well-known machine-learning technique in medical applications because it is similar to everyday problems. A classification algorithm builds a model based on training data and then applies it to test data to obtain a prediction. ^10^

Interestingly, some studies have utilized machine learning applications to predict side effects, reactogenicity, and morbidity incidence following COVID-19 vaccinations. In research by Sujatha *et al*.,, ^11^ the authors develop a model to predict whether a candidate is suitable for COVID-19 vaccination. In this paper, four machine learning approaches named Logistic Regression, AdaBoost, Random Forest, and Decision Tree were employed in the task of prediction. The authors found that AdaBoost was the classifier with the best performance, with 0.98 accuracies.

In research by Hatmal. M *et al.,* ^12^ the authors used machine learning and ensemble methods to predict the severity of side effects, defined as no, mild, moderate, or severe side effects. The analysis revealed that random forest and XGBoost achieve the highest accuracy (0.80 and 0.79, respectively) and Cohen’s κ values (0.71 and 0.70, respectively). Statistical data analysis has revealed that the side effects were significantly different based on vaccine type. According to this study, the COVID-19 vaccine that the centers for disease control and prevention (CDC) has approved is safe, and vaccination provides people with a sense of safety. However, a severe case may need additional medical care or even hospitalization.

In research by Lian *et al.,* ^13^ the goal was to collect and analyze tweets about the COVID-19 vaccination to find posts about personal experiences with COVID-19 vaccine adverse events. The authors found that the ensemble model-based RF achieves the best performance with an F1 score of 0.926, an accuracy of 0.908, and a recall of 0.946. The named entity recognition (NER) model achieved an F1 score of 0.770 for detecting adverse events using the conditional random fields (CRF) algorithm. Also, the results show that the three COVID-19 vaccines' (Pfizer, Moderna, and Johnson & Johnson) most common side effects are soreness to touch, fatigue, and headache.

## Methods

**Figure 1.**
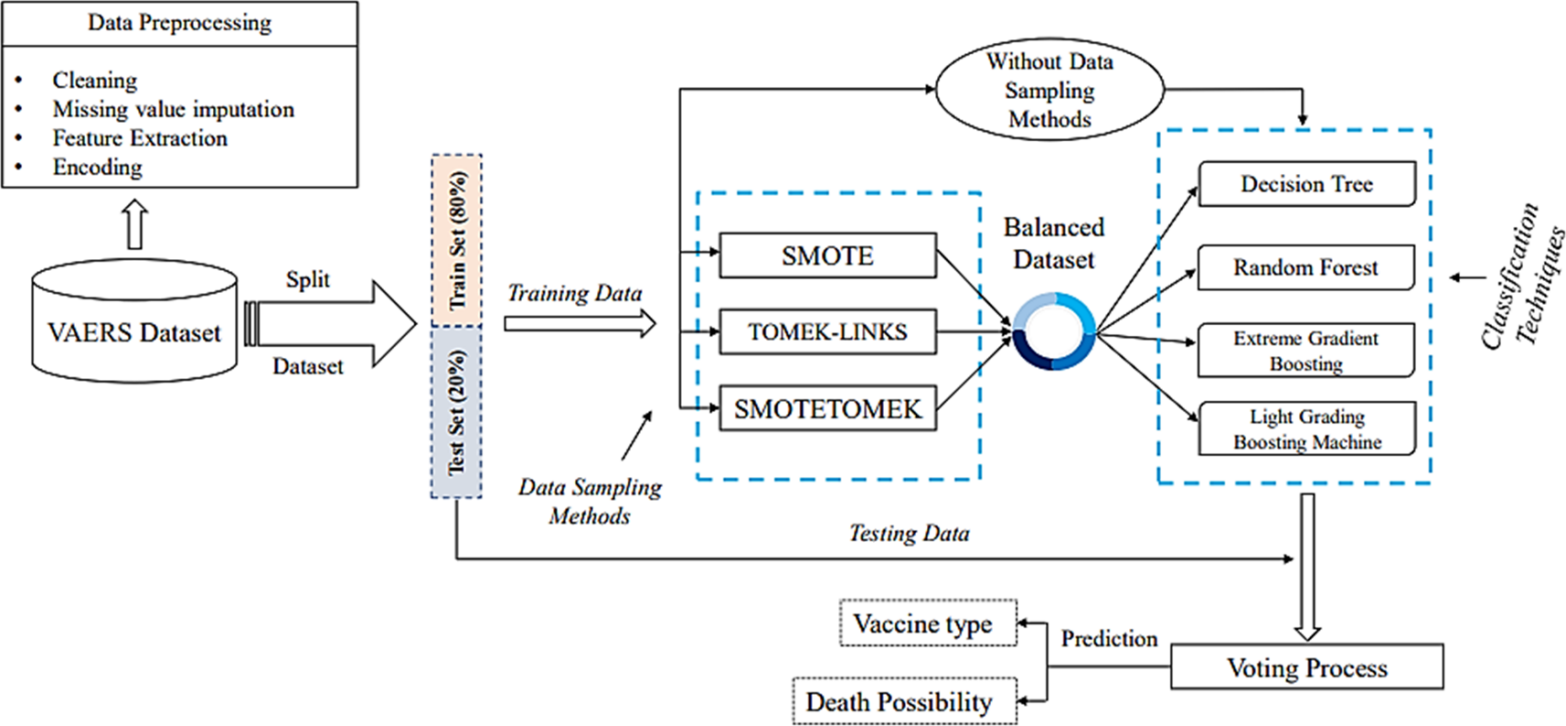
Prediction methodology architecture.

The overview of the general methodology for developing a machine learning models is visualized in Figure 1. In this study, we focus on predicting which vaccine will be most effective for a candidate without causing severe adverse reactions (output) based on several factors (input) and handling the imbalanced data that falls under the Pre-processing step where the data preparation process takes place.

### Dataset

The raw data of individuals who received vaccinations and reported adverse reactions was obtained from the VAERS. ^14^ This dataset contains vaccination information for individuals vaccinated against a variety of diseases including COVID-19, Polio, Tetanus, and Influenza. However, our current study omitted any non-SARS-CoV-2 (COVID-19) vaccination information. Therefore, the dataset being used consists of 49,810 individuals. This dataset has various attributes of individuals’ information such as age, gender, current illness, medical history, allergic history, type of vaccine, life-threatening illness, symptoms after vaccinations, etc. Some of these attributes have been found to be textual (e.g., medical history, symptoms text, etc.), while others have been found to be numerical (such as age, number of doses, etc.). The description of some different attributes in the VAERS data set is illustrated in Table 1.

**Table 1.** Description of some attributes in the VAERS dataset.

Number	Features	Description	Range	Mean	Standard Deviation
1	AGE_YRS (AS)	Age in years	16-109	57.13	18.43
2	SEX (S)	Sex information: (0: Female, 1: Male)	0-1	56.12	229.37
3	OTHER_MEDS (OM)	Other medications currently being taken	0-1	0.46	0.49
4	CUR_ILL(CL)	Illnesses at the time of vaccination	0-1	0.26	0.43
5	PRIOR_VAX(PV)	Any prior vaccination information	0-1	0.02	0.14
6	VAX_NAME (VN)	Vaccination name	0-2	0.46	0.49
7	Medical History (MH)	Pre-existing chronic or long-standing health conditions	0-1	0.46	0.49
8	ALLERGIES (A)	Any allergy history	0-1	0.25	0.43
9	Died (D)	Died	0-1	0.14	0.35

### Preprocessing

The quality of raw data used to perform any analysis heavily influences its outcome. Therefore, the preprocessing and exploratory analysis of data becomes the most important parts of any data-driven investigation. The preprocessing of a dataset involved examining the data for missing values, irrelevant values, replicas, etc. whereas EDA assists in understanding data by visualizing it. It has been noticed that the dataset contains many missing and irrelevant values.

Any COVID-19 vaccine types that were not specified were removed, and only two types of values in the sex field were considered: “M” as male and “F” as female. Unknown values were excluded. In the died field, ‘Y’ was considered yes, and the rest were considered ‘no’; in the ‘prior vaccine’ field, ‘yes’ was considered yes, and the rest were considered ‘no’. The analysis of allergic history included considering mentioned allergic effects as positive cases and considering ‘null’, ‘none’, ‘NA’, and other negatively mentioned text as negative cases. The History column in the dataset contained written records of coexisting conditions, requiring the extraction of all of the patient's medical history separately. To better understand the patient's medical history, information about pre-existing chronic and non-chronic diseases, such as chronic obstructive pulmonary disease, hypertension, diabetes, and kidney disease, was extracted. All missing values (i.e., empty, null) were excluded from this field, and spelling/grammar mistakes were fixed.

**Figure 2.**
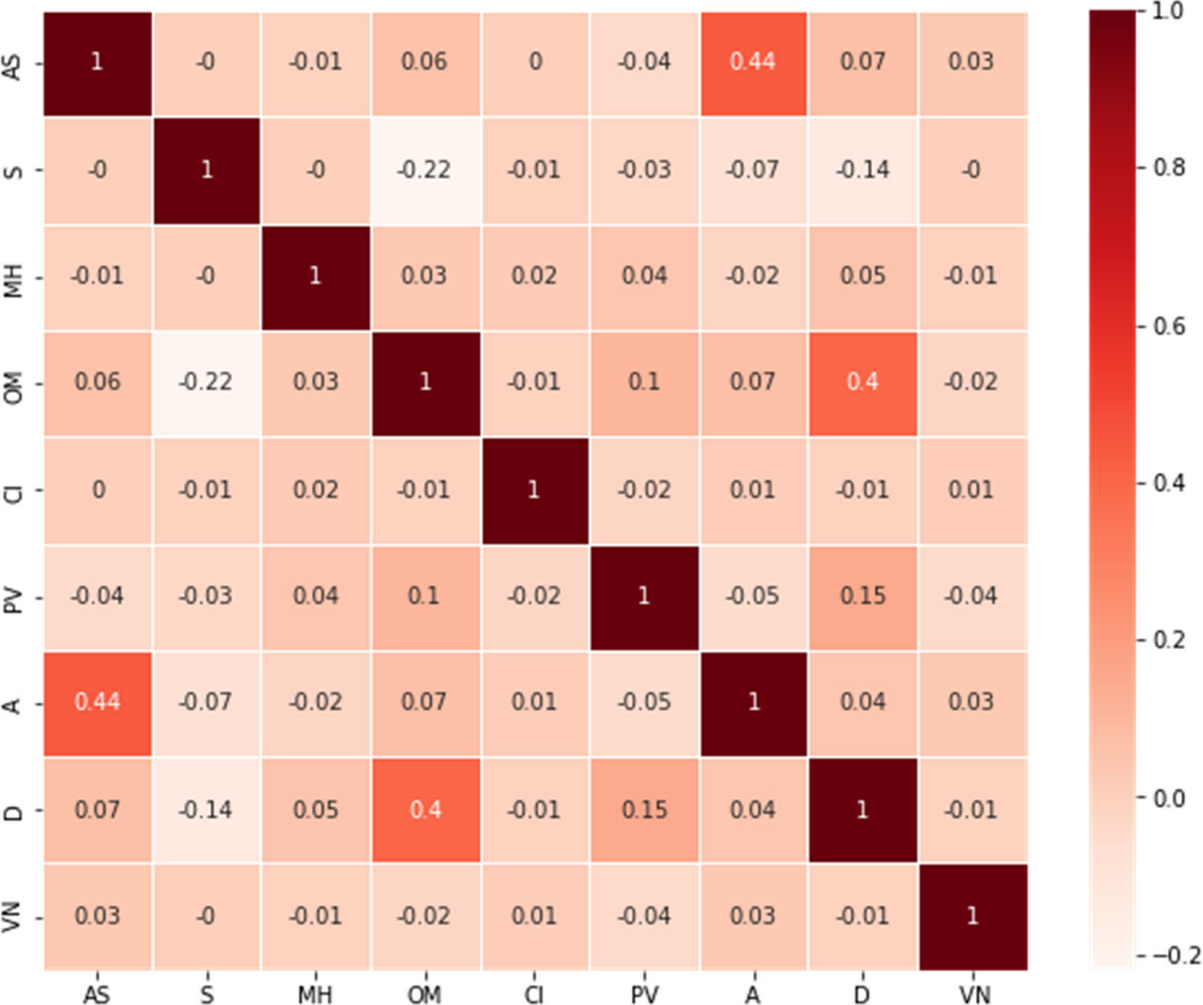
Correlation plot between different features of the VAERSA dataset.

In the Feature extraction step, most of the important features in the acquired dataset are presented as textual data. However, in order to analyze them, they must be separated into separate entities. As a result, String matching was used to convert all text data into attributes. The correlation plot ( Figure 2) did not demonstrate a significant relationship between various attributes and vaccine types. Yet, previous studies revealed a direct correlation between vaccine adverse reactions and medical and allergic histories. Therefore, the number of unique entries for the diseases in the patient's medical histories was counted. Diseases with more than 300 counts in patients' medical histories were considered attributes, while the rest were ignored due to the large dataset and the computational burden associated with each individual disease. This study, therefore, considered 21 diseases which are diabetes mellitus, thyroid, different pain, obesity, migraine, kidney disease, hypertension, hyperlipidemia, high cholesterol, heart disease, Gastroesophageal Reflux Disease (GERD), depression, dementia, positive history of COVID-19, Chronic Obstructive Pulmonary Disease (COPD), cancer, atrial fibrillation, asthma, arthritis, anxiety, and anemia from the patient’s medical history as attributes. Using the VAERS id, these files have been merged into one file after identifying and extracting features. The analyzed dataset has 28 different features and over 49,810 samples. The data was encoded using a one-hot encoding technique.

### Data-Sampling Algorithms

In this study, only three methods of handling imbalanced data are used. In the first place, no changes are made to the data. Normally, it is divided into training and testing data at a ratio of 8 to 2. This first technique is referred to as “Normal” in this study. Next, experiments are conducted using well-known imbalanced data techniques called SMOTE, Tomek-links, and SMOTETOMEK, for balancing the dataset which combines SMOTE and Tomek links. ^15^ As with the previous experiment, the dataset is divided into training and testing data at a ratio of 8 to 2. This experiment aims to handle imbalanced data and further improve the performance of machine learning classification models, especially in the multiclass classification scenario.

### Description of Ensemble Methods 

To predict which vaccine will be most effective for a candidate without causing severe adverse reactions (output) based on several factors (input), different machine-learning algorithms were used to build the proposed model.

Random Forest (RF)

A multipurpose data mining approach for classification. It is based on decision trees that operate as an ensemble, an approach of combining multiple classifiers to identify problems and enhance accuracy. A classification is predicted by each tree independently, and votes for the relevant class, and the majority of votes decide the model’s prediction. It can handle large dataset with high dimensionality, it also improves the accuracy of the model and eliminates the overfitting problem. ^16^

Decision Tree (DT)

A DT is a supervised learning technique that can be used for classification and regression problems; however, it is most commonly used to resolve classification issues. In this tree-organized classifier, the internal nodes represent datasets, branches represent decision rules, and each leaf node represents the outcome. A DT has two nodes: the decision node and the leaf node. The leaf nodes are the result of such decisions and they do not have any extra branches, but decision nodes are frequently used to settle any decision and have several branches. Based on the features of the dataset, decisions or tests are made. ^17^

Extreme Gradient Boosting (XGB)

XGBoost is an ensemble learning method combining multiple weak models' predictions to generate a stronger prediction. In the beginning, XGB fits the data to a weak classifier. Afterward, the data is fitted to another weak classifier to increase accuracy without affecting the current model. In the same way, the process continues until the best accuracy is achieved. ^18^ Furthermore, XGBoost supports parallel processing, making it possible to train models on large datasets in a reasonable period of time.

Light Grading Boosting Machine (LGBM)

LGBM is an open-source gradient boosting algorithm based on a tree-based learning framework; it is an open-source GBDT algorithm designed by Microsoft Research Asia. This framework grew trees vertically (leaf-wise) rather than horizontally (level-wise) as other tree-based frameworks did. Therefore, it can reduce the losses more efficiently and handle huge dataset with less computational complexity due to its lighter version. ^19^

### Model performance evaluation 

Macro average and Weighted Average are used to calculate the performance of the four classifiers used for learning.

• **Accuracy:** This term tells us how many classifications were correct out of all classifications.
$$\text{Accuracy}=\frac{\mathrm{TP}+\mathrm{TN}}{\mathrm{TP}+\mathrm{TN}+\mathrm{FP}+\mathrm{FN}}$$
• **Precision:** A model's precision tells us how reliable its predictions are.
$$\text{Precision}=\frac{\mathrm{TP}}{\mathrm{TP}+\mathrm{FP}}$$
• **Recall:** The model's ability to detect class.
$$\text{Recall}=\frac{\mathrm{TP}}{\mathrm{TP}+\mathrm{FN}}$$
• **F-score:** It will give us a harmonic mean of precision and recall.
$$\;F-\text{score}=2.\left(\frac{\text{precision}\cdot \text{recall}}{\text{precision}+\text{recall}}\right)$$
• ROC Curve & AUC

ROC Curves show the performance of the classification model across all classification thresholds. In a ROC curve, the TP rate and FP rate are plotted at each threshold of classification. “AUC” stands for “Area Under the ROC Curve”. It can be used as a classifier to distinguish between classes. In general, the higher the AUC value, the better the classifier is at identifying positive from negative classes. ^20^^,^^21^

## Results and Discussion

**Figure 3.**
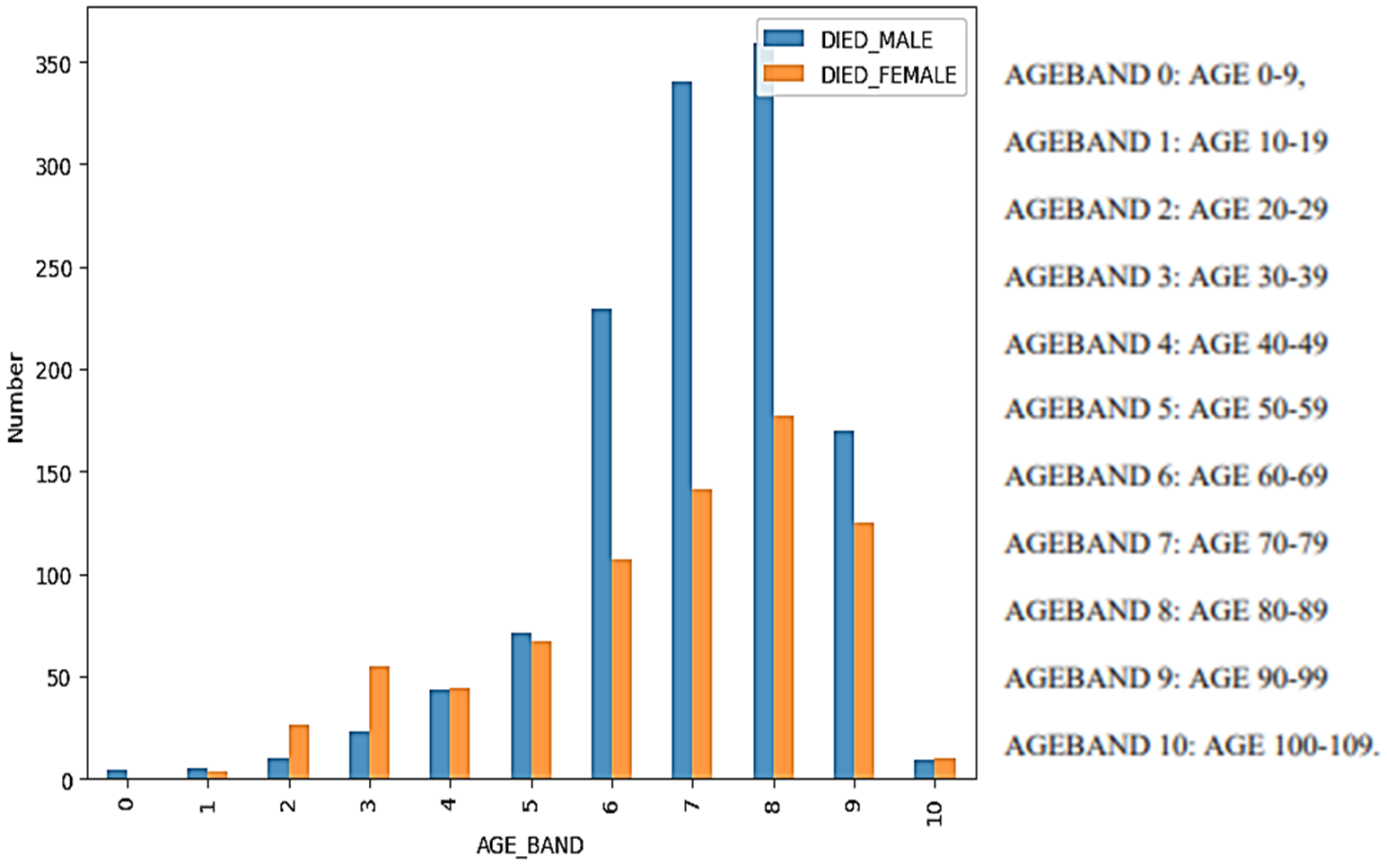
Case Fatality Number by Age Band and sex.

**Figure 4.**
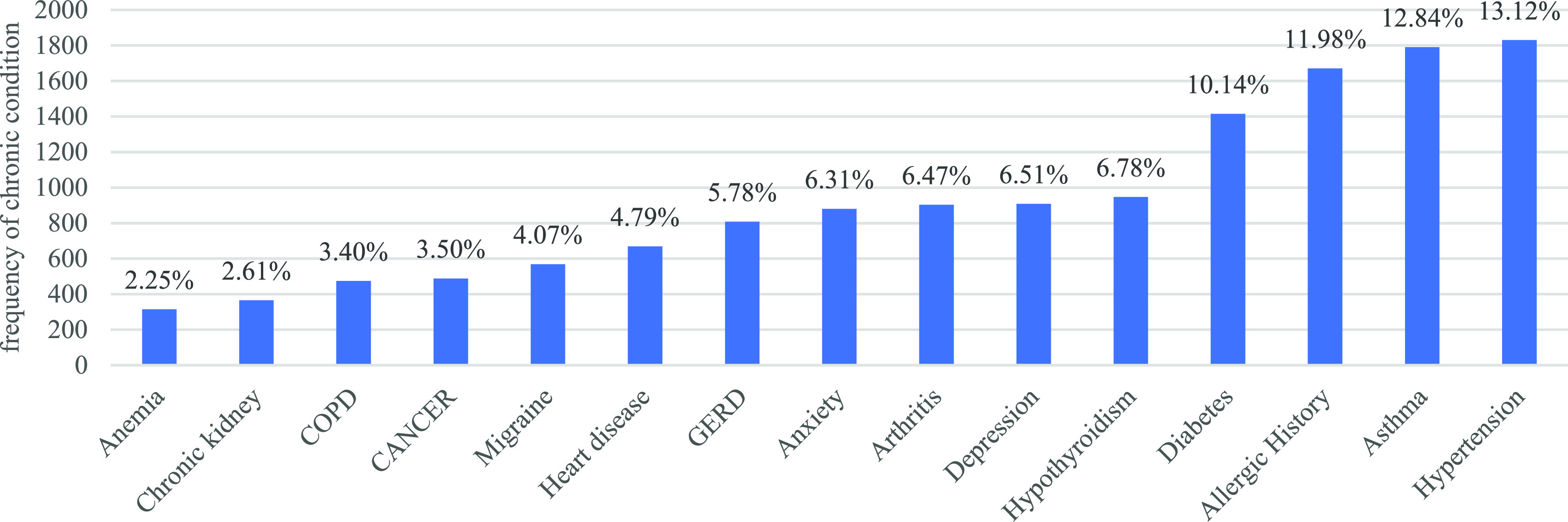
Reported chronic diseases.

The majority of the individuals, 74% in total, were identified as female It was estimated that the average age of the individuals was about 53 years old and that the average age of those who died was about 72 years. Thus, there is a noticeable age difference between the two groups. Regarding reported chronic diseases, chronic hypertension held the highest prevalence (13%), followed by asthma (12%), and kidney and anemia (2%) ( Figure 4). Among those who experienced adverse effects from vaccination, 10.7% lost their lives. From ( Figure 3), one can clearly observe that in both genders, the majority of case fatalities occurred in individuals between the ages of 70 and 89. It should also be noted that the mortality rate of males is significantly higher than that of females from the age of 60 to 99 years. Hence, men are suffering from more significant adverse effects that lead to death.

Extensive experiments have been conducted to predict three significant events in COVID-19 vaccination according to different scenarios. ML’s most relevant model to classify vaccines in each scenario includes RF, DT, XGB, and LGBM. We used 80% training data and 20% test data to evaluate the effectiveness of different ML-based approaches. As was previously mentioned, this dataset was unbalanced; therefore, We employed sampling strategies to address this problem. A number of well-known performance measures were used to assess the results of classification, including accuracy, precision, recall, F1 score, and ROC-AUC.

Our results are presented in two parts each with two scenarios: *(a)* multiclass classification with sampling, *(b)* binary classification with sampling, and *(c)* a comparison of the best model for each part.

### Multiclass classification results: based upon both medical history and vaccine type

**Figure 5.**
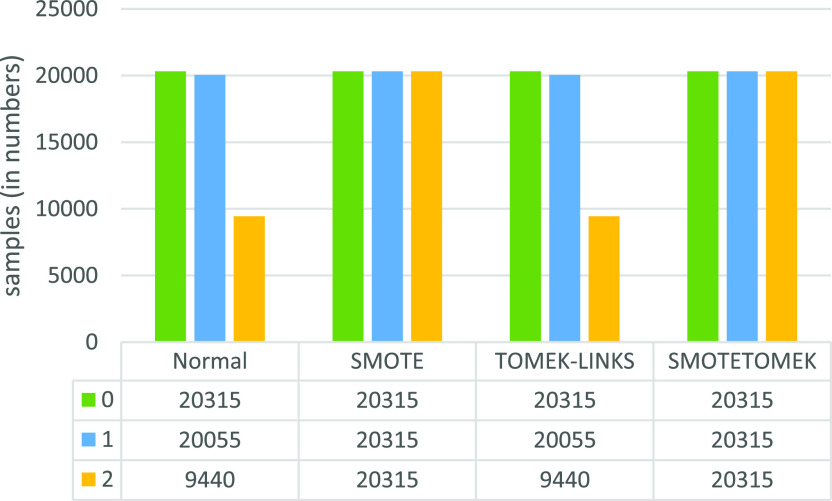
Class label counts before and after applying the various data-sampling techniques for the vaccine type dataset.

This section presents the results of the multiclass classification for covid-19 vaccine predicting problem, along with the analysis and the discussion. Firstly, we considered the patient’s medical history as independent features and the vaccine type (value 0 means Moderna, 1 means Pfizer, and 2 means Janssen) as dependent features that depend on the independent features. Then each of the three data-sampling procedures—SMOTE, TOMEK-LINKS, and SMOTETOMEK—was applied separately. ( Figure 5) illustrates the effects of applying various data-balancing techniques.

The performance parameters for each model on the test dataset are presented in ( Table 2). As a result, the following observations have been noted:

• The testing accuracy values range from approximately 75% to 81% across different models and methods. The Random Forest (RF) models with Normal, TOMEK-LINKS, and SMOTETOMEK methods achieved the highest testing accuracy of around 80.8%, while the XGBoost (XGB) and LightGBM (LGBM) models with Normal, SMOTE, and TOMEK-LINKS methods achieved slightly lower testing accuracy, ranging from 75.2% to 76.2%.
• The training accuracy values are relatively close to the testing accuracy values, indicating that the models are not overfitting to the training data. The training accuracy values range from approximately 76.9% to 81.2%.
• Macro Precision, Recall, and F1 Scores: These metrics provide insights into the models' performance for each class, and the macro averaging considers all classes equally. The RF and DT models consistently show similar precision, recall, and F1 scores across different methods, ranging from around 78.9% to 81.6%. The XGB and LGBM models tend to have slightly lower scores, ranging from approximately 70.5% to 74.6%. The RF models generally achieve the highest scores, while the XGB and LGBM models have the lowest scores.
• The AUC (Area Under the Curve) values represent the performance of the models in terms of their ability to rank samples correctly across all classes. The AUC values range from approximately 78% to 85%. The RF models with SMOTE and SMOTETOMEK methods achieved the highest AUC values of around 85%, indicating better overall performance in distinguishing between different vaccine types.
• Overall, the RF models consistently perform well across different methods, with relatively higher accuracy, precision, recall, F1 scores, and AUC values. The XGB and LGBM models have lower performance compared to RF and DT models. The SMOTE and SMOTETOMEK methods generally improve the performance of the models, as seen in higher AUC values compared to the Normal and TOMEK-LINKS methods. These models achieve relatively high testing accuracy, balanced precision, recall, and F1 scores, as well as high AUC values.

**Table 2.** Performance measures of multiclass classification.

Method	Model	Testing Accuracy	Training Accuracy	Macro Precision	Macro Recall	Macro F1 scores	AUC
Normal	RF	0.80823	0.81208	0.81569	0.78993	0.79653	0.84
	DT	0.80823	0.81210	0.81569	0.78993	0.79653	0.84
	XGB	0.76174	0.76480	0.78625	0.72301	0.74137	0.80
	LGBM	0.75218	0.75926	0.77682	0.70460	0.72229	0.78
SMOTE	RF	0.80317	0.80393	0.80226	0.80057	0.79300	0.85
	DT	0.80374	0.80397	0.80180	0.80102	0.79356	0.85
	XGB	0.75740	0.75748	0.74593	0.75048	0.74586	0.81
	LGBM	0.74953	0.75284	0.73641	0.74317	0.73613	0.80
TOMEK-LINKS	RF	0.80823	0.81208	0.81569	0.78993	0.79653	0.84
	DT	0.80823	0.81210	0.81569	0.78993	0.79653	0.84
	XGB	0.76174	0.76480	0.78625	0.72301	0.74137	0.80
	LGBM	0.75218	0.75926	0.77682	0.70460	0.72229	0.78
SMOTETOMEK	RF	0.80358	0.80451	0.80189	0.80089	0.79341	0.85
	DT	0.80374	0.80451	0.80180	0.80102	0.79356	0.85
	XGB	0.75901	0.75789	0.74595	0.75350	0.74740	0.82
	LGBM	0.75708	0.76176	0.74552	0.75530	0.74652	0.82

**Figure 6.**
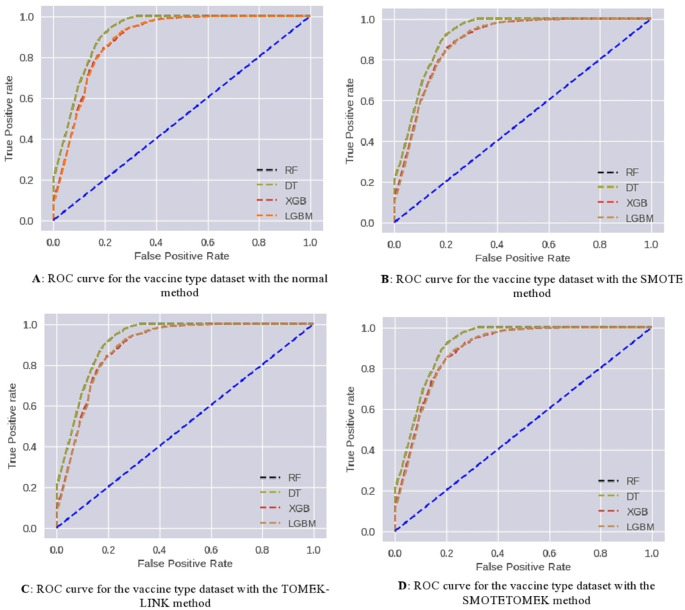
ROC curves for covid-19 type multiclass classification.

ROC curves have been used to further analyze the predictive capability of these developed models, which are shown in ( Figure 6). The RF and DT models prove their effectiveness. Taking AUC into account, all developed models perform satisfactorily.

### Binary classification results

In our model’s analysis, firstly, we considered the patient’s medical history as the independent features, and the vaccine type (value 0 means Moderna and value 1 means Pfizer) and the patient death (value 0 mean alive, and value 1 mean died) as dependent features. We trained and evaluated our models using test data by measuring accuracy, precision, recall, and AUC.

Scenario 1: Based upon both medical history and vaccine type

The performance parameters for each model on the test dataset are presented in ( Table 3). As a result, the following observations have been noted:

1. RF achieved high testing accuracy (0.87091) and training accuracy (0.87439), indicating good generalization and low overfitting. It demonstrated high precision (0.87974), recall (0.87091), and F1 score (0.87424), suggesting a balanced performance between identifying positive and negative instances. The AUC (0.93) indicates a high discriminatory power of the model. The precision value for both RF and DT was reported as 0.87. XGB and LGBM also show a comparable precision value of 0.86 and 0.0.84, respectively.
2. DT achieved similar testing accuracy (0.86975) and training accuracy (0.87439) as RF. It showed slightly lower precision (0.8779), recall (0.86975), and F1 score (0.8728) compared to RF. The AUC (0.93) suggests a good ability to distinguish between positive and negative instances.
3. XGB achieved a slightly lower testing accuracy (0.85905) and training accuracy (0.86122) compared to RF and DT. It demonstrated comparable precision (0.86031), recall (0.85905), and F1 score (0.8596) to the testing accuracy, indicating a balanced performance. The AUC (0.91) suggests a reasonably good ability to discriminate between positive and negative instances.
4. LGBM showed the lowest testing accuracy (0.84953) and training accuracy (0.85038) among the models. It had slightly lower precision (0.84771), recall (0.84953), and F1 score (0.84857) compared to the other models. The AUC (0.89) suggests a good ability to distinguish between positive and negative instances, although it is lower than RF and DT.
5. The RF and DT models with vaccine-type target consistently achieved the highest accuracy, Recall, Precision, F1 score, and AUC, especially RF outperforms all others. XGB and LGBM models had slightly lower performance metrics but still maintained reasonable accuracy and AUC.
6. Thus, the experimental analysis recommends the RF model is the most suitable for detecting vaccine type compared to the other models.

**Table 3.** Experimental performance of Scenario 1 the models with binary vaccine type dataset.

Method	Model	Testing Accuracy	Training Accuracy	Precision	Recall	F1 scores	AUC
Normal	RF	0.87091	0.87439	0.87974	0.87091	0.87424	0.93
	DT	0.86975	0.87439	0.8779	0.86975	0.8728	0.93
	XGB	0.85905	0.86122	0.86031	0.85905	0.8596	0.91
	LGBM	0.84953	0.85038	0.84771	0.84953	0.84857	0.89

**Figure 7.**
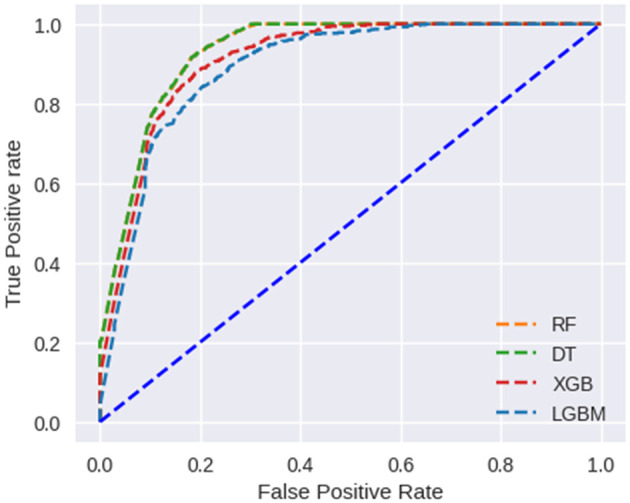
Scenario 1: ROC Curve for binary vaccine type dataset.

ROC curves have been used to further analyze the predictive capability of these models, which are shown in ( Figure 7). The RF and DT models prove their effectiveness. Taking AUC into account, all developed models perform satisfactorily.

Scenario 2: based upon both medical history and death

**Figure 8.**
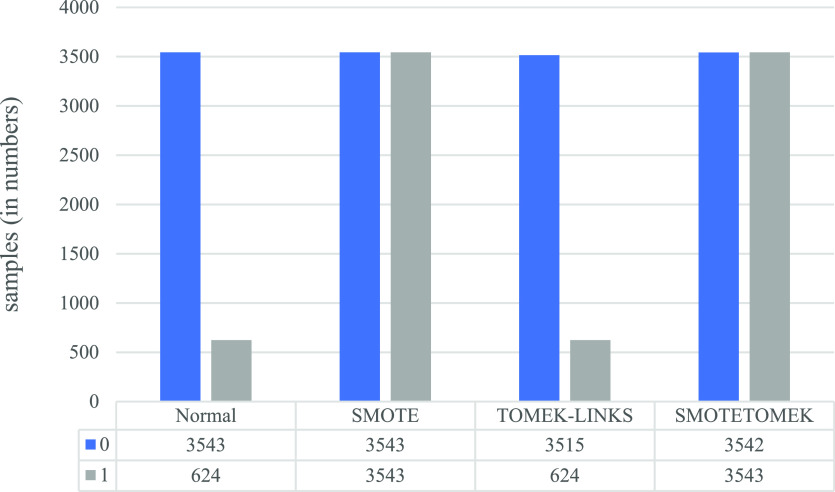
Class label counts before and after applying the various data-sampling techniques for the death dataset.

The patient’s death dataset was also experimented with as the vaccine-type dataset. ( Figure 8) demonstrates the effect of applying various data-sampling methods. The performance parameters for each model on the test dataset are presented in ( Table 4). As a result, the following observations have been noted:

1. The testing accuracy values range from approximately 79.9% to 85.7%, depending on the model and method used. The RF and XGB models consistently achieve higher testing accuracy compared to DT and LGBM models. Among the methods, TOMEK-LINKS and SMOTETOMEK methods tend to show slightly lower testing accuracy compared to Normal and SMOTE methods.
2. The training accuracy values are relatively high, ranging from approximately 87% to 95.2%. However, there is a notable difference between the training accuracy and testing accuracy values, suggesting potential overfitting issues, especially for the RF models.
3. Precision, Recall, and F1 scores: The precision, recall, and F1 scores provide insights into the models' performance for predicting the positive class (death possibility). The RF models consistently achieve higher precision, recall, and F1 scores compared to DT, XGB, and LGBM models. Among the methods, TOMEK-LINKS and SMOTETOMEK methods tend to show slightly lower precision, recall, and F1 scores compared to Normal and SMOTE methods.
4. The AUC (Area Under the Curve) values represent the models' ability to rank samples correctly and discriminate between positive and negative classes. The AUC values range from approximately 66% to 86%. The RF and XGB models consistently achieve higher AUC values, indicating better overall performance in distinguishing between COVID-19 death possibilities.
5. the models trained on the normal data generally performed better in terms of accuracy and AUC compared to the models trained on the modified datasets (SMOTE, TOMEK-LINKS, SMOTETOMEK). The Random Forest, XGBoost, and LGBM models consistently showed good performance across the metrics in all datasets, indicating their robustness and effectiveness in classification tasks. The Decision Tree model had relatively lower performance, especially in terms of AUC, in all methods.

**Table 4.** Performance measures of different methods with death dataset.

Method	Model	Testing Accuracy	Training Accuracy	Precision	Recall	F1 scores	AUC
Normal	RF	0.84261	0.95272	0.82632	0.84261	0.83277	0.83
	DT	0.82917	0.95272	0.81553	0.82917	0.82151	0.66
	XGB	0.85700	0.93064	0.83523	0.85700	0.84072	0.86
	LGBM	0.85700	0.91312	0.83938	0.85700	0.84503	0.86
SMOTE	RF	0.81861	0.92647	0.84585	0.81861	0.82961	0.83
	DT	0.81285	0.92647	0.83576	0.81285	0.82256	0.70
	XGB	0.79942	0.88992	0.84183	0.79942	0.81556	0.81
	LGBM	0.806147	0.87468	0.85443	0.80614	0.82339	0.82
TOMEK-LINKS	RF	0.84261	0.95240	0.82632	0.84261	0.83277	0.83
	DT	0.83301	0.95240	0.82065	0.83301	0.82608	0.67
	XGB	0.84932	0.92920	0.83149	0.84932	0.83791	0.85
	LGBM	0.85508	0.92002	0.83641	0.85508	0.84236	0.86
SMOTETOMEK	RF	0.80326	0.9198	0.84520	0.80326	0.81909	0.82
	DT	0.78406	0.91983	0.82101	0.78406	0.79924	0.65
	XGB	0.79846	0.88383	0.84462	0.79846	0.81563	0.81
	LGBM	0.79846	0.86958	0.84880	0.79846	0.81665	0.82

**Figure 9.**
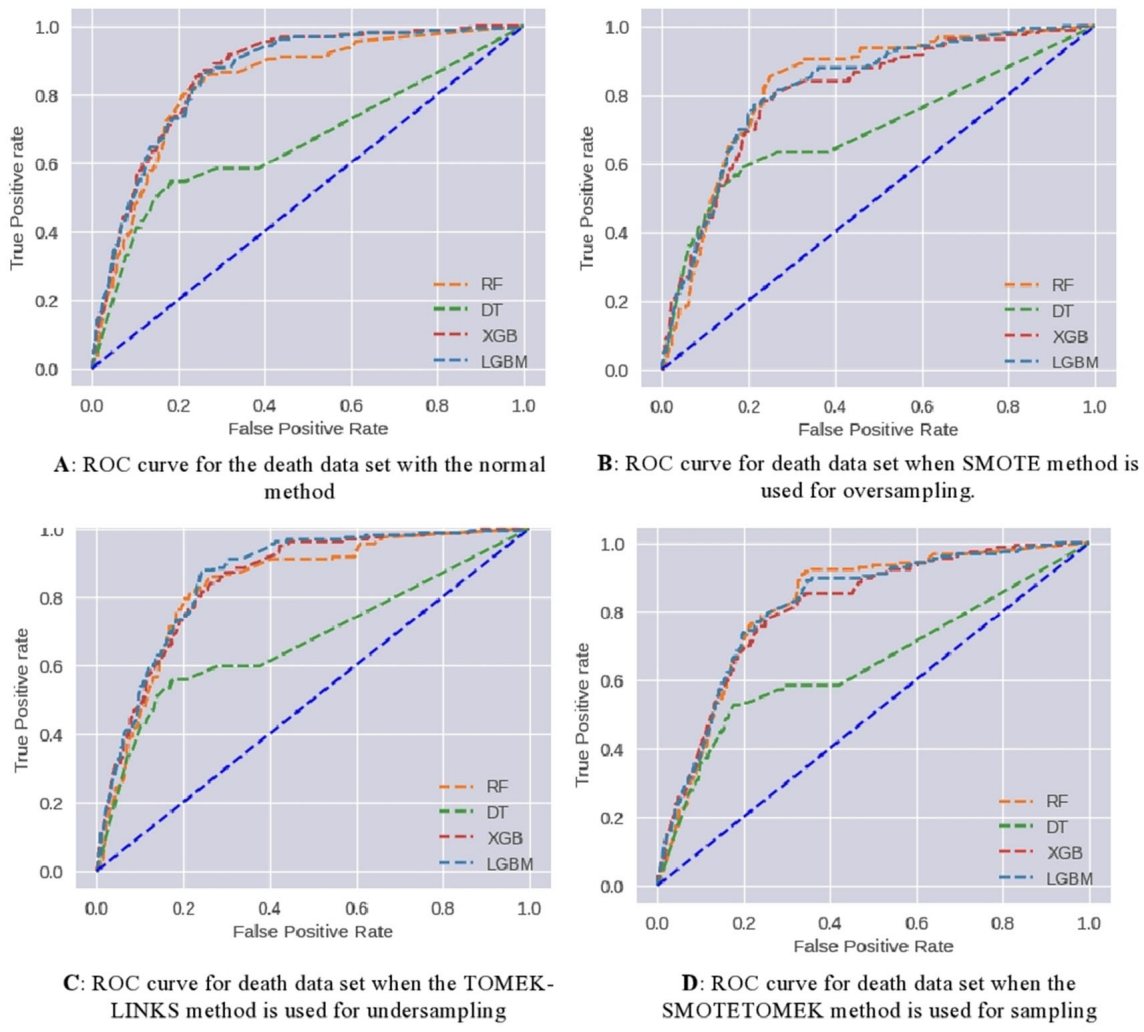
Scenario 2: ROC curve for a Death.

ROC curves have been used to further analyze the predictive capability of these models, which are shown in ( Figure 9). The RF and DT models prove their effectiveness. Taking AUC into account, all developed models perform satisfactorily.

**Figure 10.**
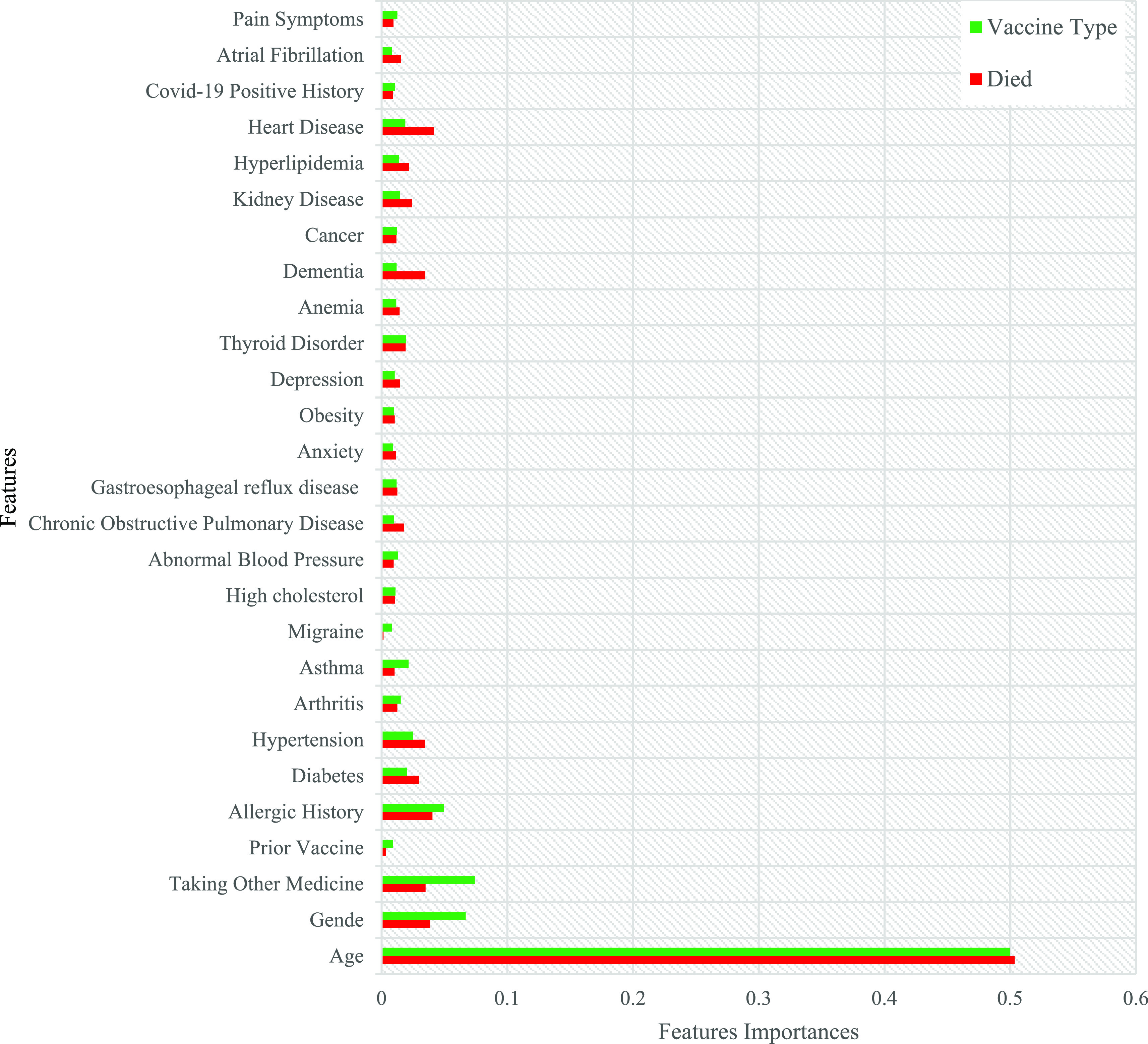
Ranking of features based on the patients' medical history coefficient values.

The importance of all the features in the COVID-19 vaccine adverse reactions dataset is calculated using the feature_importance package from the Scikit-learn Python library. A visual representation of the calculated values for feature importance is displayed in Figure 10. The features are arranged based on their respective importance scores.

Figure 10 shows that patients' age, gender, and use of other medicines were significant factors in the past medical history of all target variables. WHEN examining the target variable of “vaccine type,” the analysis revealed a comprehensive set of critical attributes within the patient's medical history that strongly influence the selection of the administered vaccine. These attributes include previous vaccine history, allergic history, diabetes, arthritis, hypertension, and asthma. Furthermore, when investigating the target variable of death status, certain factors emerged as highly significant. These factors include heart disease, allergic history, dementia, hypertension, diabetes, kidney disease, and Chronic obstructive pulmonary disease (COPD). These attributes have shown a noteworthy impact on the desired outcome, indicating their importance in predicting the death status of patients.

The patient's age and gender provide essential demographic information that may impact the choice of vaccine, as certain vaccines have age or gender-specific recommendations. Additionally, considering the patient's current medication usage is crucial to ensure compatibility and potential interactions with the chosen vaccine. Previous vaccine history helps determine if the patient requires a booster or a specific type of vaccine.

The presence of underlying conditions such as diabetes, arthritis, allergic history, hypertension, and asthma is highly influential in the decision-making process. These conditions may affect the patient's immune response or make them more susceptible to certain vaccine side effects. By considering these attributes, healthcare professionals can tailor the vaccine type to maximize efficacy and minimize risks for each patient.

### Strengths and limitations

As far as the authors are aware, this is the first study that attempts to predict the type of covid-19 vaccine appropriate for a candidate, along with the death probability risk. Additionally, we suggest approaches to address the issue of imbalanced data concerning adverse reactions to COVID-19 vaccines.

This study has some limitations. Because these data were collected online, we cannot rule out information-gathering bias in the study. Moreover, this data set contained a significant amount of missing data, which may lead to a misrepresentation of patient populations.

## Conclusion and future works

In this work, four ML models were evaluated: DT, RF, XGBoost, and LGBM. Three sampling techniques were executed for each model to handle imbalanced data. Below are some of the key findings of the study, which shed light on crucial insights and implications:

1. The tree-based model RF presented the best overall results with multiclass classification.
2. The SMOTE and SMOTETOMEK methods generally improve the performance of the models, as seen in higher AUC values compared to the Normal and TOMEK-LINKS methods.
3. For binary classification in scenario 1, the experimental analysis recommends the RF model as the most suitable for detecting vaccine type compared to the other models.
4. In scenario 2, the RF, XGBoost, and LGBM models consistently showed good performance across the metrics in all methods, indicating their robustness and effectiveness in classification tasks.
5. The Decision Tree model had relatively lower performance, especially in terms of AUC, in all methods.
6. The results revealed that patient age, gender, allergic history, prior vaccine, other medicines, diabetes, hypertension, and heart disease are significant pre-existing factors that strongly influence the selection of the administered vaccine.

According to the study's results, the RF model is recommended for machine learning tasks that demand high accuracy and robustness. While both the XGBoost and LGBM models are also viable options, the RF model could be preferable when dealing with imbalanced data.

This work can also be applied to any other datasets related to vaccinations. we limited the number of medical history features due to the large dataset size and the computational burden associated with processing each disease. However, further advancements can be made by automating the system to analyze predictions based on more medical history features. As new data is entered into the dataset, the automation can generate new predictions based on the prevailing factors at that particular moment. Furthermore, deep learning models can also be used to extract more hidden patterns to improve COVID-19 vaccine acceptability by better understanding its dynamics.

## Data Availability

The dataset used to support the findings of this study is available at the following: https://vaers.hhs.gov/data/datasets.html. The dataset is comprised of three CSV files, namely VAERSDATA, VAERSVAX, and VAERSSYMPTOMS. Within these datasets, VAERSDATA provides comprehensive information regarding individuals, VAERSVAX offers details related to vaccines, encompassing vaccination type, manufacturer, dosage count, and vaccination location, and VAERSSYMPTOMS catalog symptoms reported as various illnesses following vaccinations. [VAERS Data]: https://vaers.hhs.gov/eSubDownload/index.jsp?fn=2021VAERSDATA.csv. [VAERS Vaccine]: https://vaers.hhs.gov/eSubDownload/index.jsp?fn=2021VAERSVAX.csv. [VAERS Symptoms]: https://vaers.hhs.gov/eSubDownload/index.jsp?fn=2021VAERSSYMPTOMS.csv.
