# Supplementary material for: COVID-19 Vaccine: Predicting Vaccine Types and Assessing Mortality Risk Through Ensemble Learning Algorithms
Source: F1000Res. 2024 Aug 23;12:1200. Originally published 2023 Sep 25. [Version 2] doi: 10.12688/f1000research.140395.2 (PMC11128056; doi:10.12688/f1000research.140395.2)
Supplement: Peer review history

# Reviewer report: Reviewer response for version 2

**DOI:** 10.5256/f1000research.167975.r316661

**Published:** 2024-09-05

**Authors:** Samir Kumar Bandyopadhyay

**Affiliations:** 1 The Bhawanipur Education Society College, Kolkata, India

After reviewing the revision made by the authors I am satisfied with the revision.

Is the work clearly and accurately presented and does it cite the current literature?

No

If applicable, is the statistical analysis and its interpretation appropriate?

Yes

Are all the source data underlying the results available to ensure full reproducibility?

Yes

Is the study design appropriate and is the work technically sound?

Yes

Are the conclusions drawn adequately supported by the results?

No

Are sufficient details of methods and analysis provided to allow replication by others?

No

Reviewer Expertise:

Image Processing, Forensic Science, Machine Learning, Crime Science, Data Science, etc.

I confirm that I have read this submission and believe that I have an appropriate level of expertise to confirm that it is of an acceptable scientific standard.

# Reviewer report: Reviewer response for version 1

**DOI:** 10.5256/f1000research.153740.r233060

**Published:** 2024-05-31

**Authors:** Samir Kumar Bandyopadhyay

**Affiliations:** 1 The Bhawanipur Education Society College, Kolkata, India

Authors:

 Q1. Specify the imbalanced dataset with an example.

 Q2.What type of Illnesses can be seen at the time of vaccination?

 Q3. Explain EDA since otherwise it is difficult to understand the pre-processing steps.

 Q4 Pre-existing chronic disease is required to study and it must contain fever nature.

 Q5.The process of feature extraction is not provided accurately.

 Q6. What is the difference between attribute values and attribute text?

 Q7. There are no comparisons with existing methods.

 Editor:

 Authors studied to predict which vaccine will be most effective for a candidate without causing severe adverse reactions and to categorize a patient as potentially at high risk of death from the COVID-19 vaccine. In the Feature extraction step, most of the important features in the acquired dataset are presented as textual data. However, in order to analyze them, they must be separated into separate entities. All important parts are not explained properly.

Is the work clearly and accurately presented and does it cite the current literature?

No

If applicable, is the statistical analysis and its interpretation appropriate?

Yes

Are all the source data underlying the results available to ensure full reproducibility?

Yes

Is the study design appropriate and is the work technically sound?

Yes

Are the conclusions drawn adequately supported by the results?

No

Are sufficient details of methods and analysis provided to allow replication by others?

No

Reviewer Expertise:

Image Processing, Forensic Science, Machine Learning, Crime Science, Data Science, etc.

I confirm that I have read this submission and believe that I have an appropriate level of expertise to state that I do not consider it to be of an acceptable scientific standard, for reasons outlined above.

# Response

**Published:** 2024-06-14

**Authors:** Hind Monadhel

**Affiliations:** Computer Science, University of Technology Baghdad, Baghdad, Baghdad, Iraq

Dear Samir,

 Thank you for dedicating your time and effort to review our manuscript. Your insightful comments and suggestions have provided valuable guidance for improving our work.

 While we are disappointed by the decision, we genuinely appreciate the constructive feedback that will undoubtedly strengthen our future research efforts.

 Thank you once again for your thorough review.

# Reviewer report: Reviewer response for version 1

**DOI:** 10.5256/f1000research.153740.r257039

**Published:** 2024-05-25

**Authors:** Dhamodharavadhani S

**Affiliations:** 1 Department of Computer Applications, ARULMIGU ARTHANAREESWARAR ARTS AND SCINCE COLLEGE, Salem, Tamil Nadu, India

Overall, this manuscript makes a significant contribution to the field of healthcare analytics by leveraging machine learning techniques to predict vaccine effectiveness and mortality risk associated with COVID-19 vaccination. The findings have important implications for improving vaccination strategies and patient care. However, I recommend some minor revisions for clarity and precision in language.

 Certainly! Here are some potential review questions for the manuscript:

 1. What are the main objectives of the study, and why are they important in the context of COVID-19 vaccination?

 2. How does the study contribute to the existing literature on vaccine effectiveness and adverse reactions?

 3. Can you explain the rationale behind selecting ensemble learning algorithms for this study?

 4. How were class balancing techniques like SMOTE, TOMEK_LINK, and SMOTETOMEK incorporated into the analysis, and why were they deemed necessary?

 5. What were the key factors identified as crucial for predicting vaccine effectiveness and mortality risk?

 6. elaborate on the performance metrics used to evaluate the ensemble learning classifiers, and how do they reflect the predictive accuracy of the models?

 7. How do the performance outcomes of different ensemble learning algorithms compare in predicting vaccine types and mortality risk?

 8. What insights do the findings provide regarding the relationship between pre-existing conditions, medical history, and vaccine outcomes?

 9. What practical implications do the study findings have for healthcare practitioners and policymakers?

 10. Based on the results obtained, what recommendations would you provide for optimizing COVID-19 vaccination strategies and minimizing adverse reactions?

 11.  Are there any limitations of the current study that should be addressed in future research?

   12. What additional research avenues do you suggest for further advancing our understanding of vaccine effectiveness and safety prediction?

 13. In your opinion, how does this study contribute to advancing knowledge in the field of healthcare analytics and COVID-19 vaccination?

 19. What are the strengths and weaknesses of the manuscript, and how could they be addressed to enhance its impact and credibility?

Is the work clearly and accurately presented and does it cite the current literature?

Yes

If applicable, is the statistical analysis and its interpretation appropriate?

Partly

Are all the source data underlying the results available to ensure full reproducibility?

Yes

Is the study design appropriate and is the work technically sound?

Partly

Are the conclusions drawn adequately supported by the results?

Yes

Are sufficient details of methods and analysis provided to allow replication by others?

Partly

Reviewer Expertise:

Machine learning, Predictive Analytics, Data Science, optimization, Big data Analytics

I confirm that I have read this submission and believe that I have an appropriate level of expertise to confirm that it is of an acceptable scientific standard, however I have significant reservations, as outlined above.

## References

[1] : Nonlinear Neural Network Based Forecasting Model for Predicting COVID-19 Cases. *Neural Process Lett* .2023;55(1) : 10.1007/s11063-021-10495-w 171-191 10.1007/s11063-021-10495-w 33821142 PMC8012519

[2] : COVID-19 Mortality Rate Prediction for India Using Statistical Neural Network Models. *Front Public Health* .2020;8: 10.3389/fpubh.2020.00441 441 10.3389/fpubh.2020.00441 32984242 PMC7485390

[3] : COVID-19 mortality rate prediction for India using statistical neural networks and gaussian process regression model. *Afr Health Sci* .2021;21(1) : 10.4314/ahs.v21i1.26 194-206 10.4314/ahs.v21i1.26 34394298 PMC8356626

[4] : SEIR Model for COVID-19 Epidemic Using Delay Differential Equation. *Journal of Physics: Conference Series* .2021;1767(1) : 10.1088/1742-6596/1767/1/012005 10.1088/1742-6596/1767/1/012005

[5] : Novel COVID-19 Mortality Rate Prediction (MRP) Model for India Using Regression Model With Optimized Hyperparameter. *Journal of Cases on Information Technology* .2021;23(4) : 10.4018/JCIT.20211001.oa1 1-12 10.4018/JCIT.20211001.oa1

[6] : Coffee leaf valorisation into functional wheat flour rusk: their nutritional, physicochemical, and sensory properties. *J Food Sci Technol* .2024;61(6) : 10.1007/s13197-024-05927-z 1117-1125 10.1007/s13197-024-05927-z 38562602 PMC10981639

[7] : Vaccine rate forecast for COVID-19 in Africa using hybrid forecasting models. *Afr Health Sci* .2023;23(1) : 10.4314/ahs.v23i1.11 93-103 10.4314/ahs.v23i1.11 37545978 PMC10398474

# Response

**Published:** 2024-06-14

**Authors:** Hind Monadhel

**Affiliations:** Computer Science, University of Technology Baghdad, Baghdad, Baghdad, Iraq

Dear Dhamodharavadhani S,

 Thank you for your valuable feedback and suggestions. We appreciate your thorough review and believe that these revisions have significantly enhanced the quality of our work.

# Reviewer report: Reviewer response for version 1

**DOI:** 10.5256/f1000research.153740.r245449

**Published:** 2024-05-03

**Authors:** Jinran (Ryan) Wu

**Affiliations:** 1 Australian Catholic University, Fitzroy, Victoria, Australia

The authors proposed predicting vaccine types and assessing mortality risk through some ensemble learning approaches. The research topic is interesting, and some comments are given for further consideration.

 1. Based on Google Scholar, authors missed citing many recent references that shouldn't be ignored. Here, I suggest using a table to list and compare their main points to highlight your contributions to the area.

 2. For the experiment part, all parameter settings are missing, so that the results declared cannot be repeated. Please release your codes or detailed settings in the appendix.

 3. For your results, I suggest authors use a cross-validation approach to evaluate the uncertainty of the predictions. 

 4. Also, considering the imbalance issue, the authors shall consider different penalties for different prediction errors. In other words, we cannot regard "dies" as the same as "non-dies". Authors shall distinguish different losses.

 5. The discussion could have been improved. The authors shall further explore the underlying implications in this part. Otherwise, the work looks like a mathematical game. In particular, the authors shall connect results to some findings from some top medicine journals.

 6. The authors shall use a professional writing service to make the content clear.

Is the work clearly and accurately presented and does it cite the current literature?

Partly

If applicable, is the statistical analysis and its interpretation appropriate?

Partly

Are all the source data underlying the results available to ensure full reproducibility?

Partly

Is the study design appropriate and is the work technically sound?

Partly

Are the conclusions drawn adequately supported by the results?

Partly

Are sufficient details of methods and analysis provided to allow replication by others?

Partly

Reviewer Expertise:

Machine learning, Forecasting, Applied Statistics

I confirm that I have read this submission and believe that I have an appropriate level of expertise to confirm that it is of an acceptable scientific standard, however I have significant reservations, as outlined above.

# Response

**Published:** 2024-06-14

**Authors:** Hind Monadhel

**Affiliations:** Computer Science, University of Technology Baghdad, Baghdad, Baghdad, Iraq

Dear Jinran Wu,

 Thank you for your valuable feedback and suggestions. We have carefully addressed all your comments and revised the manuscript accordingly. Specifically, regarding your suggestion " 3. For your results, I suggest authors use a cross-validation approach to evaluate the uncertainty of the predictions," we will upload the experimental code to ensure clarity.

 We appreciate your thorough review and believe these revisions have significantly enhanced the quality of our work.

# Reviewer report: Reviewer response for version 1

**DOI:** 10.5256/f1000research.153740.r257042

**Published:** 2024-03-28

**Authors:** Aritra Ghosh

**Affiliations:** 1 Computer Science, Sister Nivedita University, Kolkata, West Bengal, India

**Full Report:**

**Introduction:** The introduction provides a comprehensive overview of the transition in vaccine development timelines and the urgent need for effective COVID-19 vaccines. It highlights the significance of understanding vaccine mechanisms and adverse reactions, setting the stage for the proposed machine-learning framework. However, it could benefit from a succinct statement of the study's objectives to guide readers through the subsequent sections more effectively.

**Literature Review:** The literature review effectively contextualizes the study within existing research on machine learning applications in healthcare, specifically focusing on COVID-19 vaccine prediction. It provides insights into relevant studies while emphasizing the novelty and contributions of the current work. However, the review could be strengthened by discussing potential limitations or gaps in previous research, thereby justifying the need for the proposed study more explicitly.

**Methods:** The methods section is detailed and well-structured, outlining the data preprocessing, feature extraction, and modeling techniques employed. It effectively communicates the rationale behind each step and provides clarity on the experimental design. The inclusion of figures and tables enhances the understanding of complex methodologies. However, providing more information on the rationale behind the selection of specific sampling techniques and model evaluation metrics would strengthen the methodology further.

**Results and Discussion:** The results and discussion section presents comprehensive findings from the study, including performance metrics and an analysis of key features. The results are effectively communicated through tables, figures, and textual descriptions, facilitating interpretation. The discussion contextualizes the findings within the broader literature and highlights implications for vaccine selection and adverse reaction prediction. However, a more structured approach to discussing limitations and future research directions would enhance the clarity of the discussion.

**Conclusion and Future Works:** The conclusion summarizes the key findings and implications of the study while outlining potential avenues for future research. It effectively emphasizes the significance of the study's contributions and underscores the importance of continued research in this area. However, providing more specific recommendations for addressing identified limitations and mitigating potential biases would enhance the conclusion's comprehensiveness.

**Overall Assessment:** The abstract and full report provides a detailed and insightful analysis of the proposed machine-learning framework for COVID-19 vaccine prediction. The study demonstrates a rigorous approach to data analysis and model evaluation, yielding valuable insights into vaccine efficacy and adverse reactions. Addressing the following minor issues would further enhance the scientific soundness and readability of the report:

  

1. **Clarity and Readability:**
  - Ensure consistent terminology throughout the report. For example, use either "COVID-19 vaccine" or "SARS-CoV-2 vaccine" consistently instead of switching between them.
  - Consider breaking down lengthy paragraphs into shorter ones for easier readability and comprehension, especially in sections like "Results and Discussion" and "Conclusion and Future Works."
  - Provide clear transitions between sections to guide the reader through the report more effectively. Each section should flow logically from one to the next.
2. **Justification:**
  - Provide more justification for the choice of machine learning algorithms. Explain why Random Forest (RF), Decision Tree (DT), Extreme Gradient Boosting (XGB), and Light Grading Boosting Machine (LGBM) were selected over other algorithms. Justify why these algorithms are suitable for the task at hand.
  - Clarify the reasoning behind choosing specific data sampling techniques (e.g., SMOTE, Tomek-links, SMOTETOMEK) to handle imbalanced data. Explain why these techniques were deemed appropriate and how they contribute to improving model performance.
3. **Structure:**
  - Consider refining the structure of the report to make it more cohesive and organized. For instance, ensure that each section has a clear and specific focus, with subheadings to delineate different topics within the section.
  - Provide a brief overview or summary at the beginning of each section to outline the main points that will be discussed. This will help readers understand the purpose and scope of each section more clearly.
  - In the "Conclusion and Future Works" section, provide a concise summary of the key findings and implications of the study. Additionally, offers specific suggestions for future research directions based on the limitations or areas for improvement identified in the study.

By addressing these minor issues, the report will become more scientifically sound and easier to follow for readers, thereby enhancing its overall quality and impact.

Is the work clearly and accurately presented and does it cite the current literature?

Yes

If applicable, is the statistical analysis and its interpretation appropriate?

Yes

Are all the source data underlying the results available to ensure full reproducibility?

Yes

Is the study design appropriate and is the work technically sound?

Yes

Are the conclusions drawn adequately supported by the results?

Yes

Are sufficient details of methods and analysis provided to allow replication by others?

Yes

Reviewer Expertise:

Data Analysis, AI and ML, HCI, Computational Modeling and Big Data, and Web Development. Cuurently working on the application of Machine Learning for COVID-19 vaccine development.

I confirm that I have read this submission and believe that I have an appropriate level of expertise to confirm that it is of an acceptable scientific standard.

# Response

**Published:** 2024-06-14

**Authors:** Hind Monadhel

**Affiliations:** Computer Science, University of Technology Baghdad, Baghdad, Baghdad, Iraq

Dear Aritra Ghosh,

 Thank you for your valuable feedback and suggestions. We have carefully addressed all your comments and have revised the manuscript accordingly.

 We appreciate your thorough review and believe the changes have significantly improved the quality of our work.

 Hind M.Abdullah
